# Reliability and construct validity of the Italian version of AMAT scale in SBMA subjects

**DOI:** 10.1007/s10072-026-09052-x

**Published:** 2026-05-02

**Authors:** Elisabetta Pupillo, Elisa Bianchi, Maurizio A. Leone, Sabrina Paganoni, Alberto Romito, Federica Paredi, Giacomo Maria Minicuci, Gianni Sorarù

**Affiliations:** 1 Laboratory of Neurological Disorders, Department of Neuroscience, Istituto di Ricerche Farmacologiche Mario Negri IRCCS, VIA Mario Negri 2, 20156 Milano, Italy; 2 Sean M. Healey & AMG Center for ALS and Neurological Clinical Research Institute at Massachusetts General Hospital, Harvard Medical School, Boston, MA USA; 3 Spaulding Rehabilitation Hospital, Harvard Medical School, Boston, MA USA; 4 Motor Neuron Disease Clinic, Azienda Ospedale Università, Department of Neurosciences, University of Padova, Via Nicolo’ Giustiniani 2, 35128 Padova, Italy

**Keywords:** Validation, AMAT scale, Italian translation, SBMA

## Abstract

**Background:**

Spinal and Bulbar Muscular Atrophy (SBMA) is a rare X-linked polyglutamine disorder characterized by a CAG trinucleotide repeat expansion in the androgen receptor gene. This leads to progressive lower motor neuron degeneration and skeletal muscle atrophy. Given the need for sensitive outcome measures in clinical trials, this study aimed to perform the linguistic adaptation and psychometric validation of the Adult Myopathy Assessment Tool (AMAT) for the Italian population.

**Methods:**

Following a rigorous forward-back translation protocol to ensure semantic and conceptual equivalence, the Italian AMAT was administered to 29 patients. The validation process assessed internal consistency (Cronbach’s alpha), inter-rater and intra-rater reliability, and construct validity. The latter was evaluated through correlations with established clinical markers, including the Six-Minute Walk Test (6MWT), the SBMA Functional Rating Scale (SBMAFRS), and the ALSAQ-40 scale.

**Results:**

Psychometric analysis revealed excellent inter- and intra-rater reliability and strong internal consistency (Cronbach’s alpha > 0.70). Construct validity was confirmed through significant correlations with established functional markers, including the six-minute walk test (6MWT) and the SBMA Functional Rating Scale (SBMAFRS), while the expected negative correlations with ALSAQ-40 scale physical domains—coupled with a lack of correlation with the communication domain—affirmed divergent validity.

**Conclusions:**

The Italian version of the AMAT is a reliable and valid instrument for quantifying functional impairment and endurance in SBMA. Its implementation facilitates standardized longitudinal assessment and enhances the feasibility of cross-national collaborative research.

**Supplementary Information:**

The online version contains supplementary material available at 10.1007/s10072-026-09052-x.

## Introduction

Spinal and bulbar muscular atrophy (SBMA), also known as Kennedy’s disease, is a late-onset neuromuscular disorder affecting skeletal muscle and lower motor neuron degeneration in brainstem and spinal cord [1, 2]. It is caused by a trinucleotide CAG repeat expansion (CAG > 37) in the androgen receptor gene, mapping to X-chromosome, encoding for a polyQ tract [3, 4]. The disease typically manifests in males who develop proximal limb and bulbar muscle weakness along with signs of partial androgen insensitivity [5]. Female carriers may exhibit some neuromuscular features [6]. SBMA follows a slowly progressive course, and currently, no curative treatments are available.

The Adult Myopathy Assessment Tool (AMAT scale) was specifically modified by Michael O. Harris-Love et al. in 2014 [7] to evaluate both impairment and functional limitations in individuals with SBMA. Compared with other functional measures considered for SBMA, e.g. Spinal and Bulbar Muscular Atrophy Functional Rating Scale (SBMAFRS) [8], the AMAT places greater emphasis on proximal strength, postural control, and endurance. The final version of the scale comprises 13 items (sequenced to mitigate the impact of fatigue) and is categorized into two subscales: the functional AMAT subscale and the endurance AMAT subscale. Administration typically takes 25–35 min and requires only commonly available equipment.

The scale was validated in a sample of 56 SBMA subjects. It showed excellent construct validity and good internal consistency for adults with SBMA (Cronbach’s 𝛼 = 0.77–0.89; 𝑃 < 0.001). These findings were supported by significant associations with strength, objective and subjective physical performance measures, and self-reported physical status.

In an ongoing clinical trial evaluating the efficacy and safety of the β2-agonist clenbuterol in SBMA in 7 Italian centres, the AMAT scale was selected as a secondary outcome measure for patient assessment. As consequence, a translation and validation of the AMAT scale into Italian was needed in this context.

## Methods

The initial phase for the validation of the Italian version of the AMAT scale entailed a formal forward/back translation process of its original English version. First, two local investigators, both native Italian speakers independently translated the English version into Italian. Discrepancies between their translations were resolved through an in-person meeting to achieve a consensus on the final Italian text. Subsequently, this Italian text was back-translated into English by a different translator: an Italian physician who has been residing in the US for 25 years and was blinded to the initial forward step of the process. The back-translated text was then compared with the original source, identifying any discrepancies, inconsistencies, or shifts in meaning. A third Italian first language investigator reconciled differences, ensuring that the final translated version accurately reflects the original one. The final Italian version of the AMAT scale is provided in appendix 1. The Italian version of the AMAT was then administered to 29 consecutive patients. Demographics and clinical characteristics of patients were recorded. Each subject was independently assessed by the two local neurologists (GS and GMM). The first assessment was conducted at the screening visit, the second was performed at the randomization visit, which occurred 30 days later and a third assessment occurred at the 4-week follow-up visit. Considering the slow progression of SBMA, a 30-day interval was deemed insufficient to influence AMAT outcomes. For each participant, the assessments at randomization and 4-week follow-up visit were done by the same neurologist, while the assessment at the screening visit was done by the other neurologist, to be able to evaluate both inter- and intra-rater reliability. The values of the 6-min walk distance (6MWT), widely accepted as the most reliable marker of motor impairment in SBMA [9], the scores of the SBMAFRS score [8], a questionnaire measuring physical function in activities of daily living (ADL) [10], and the score of the Amyotrophic Lateral Sclerosis Assessment Questionnaire-40, (ALSAQ-40), a quality-of-life scale for ALS patients [11], were all collected at the randomization visit according to the clinical trial protocol and included in our analysis. Validated Italian translation for both SBMAFRS and ALSAQ-40 scales were used in the present study.

All subjects signed consent to the study participation.

### Statistical analysis

Descriptive statistics were performed for demographical and clinical characteristics of the sample, for the scores of functional and quality of life scales. Continuous variables were described by medians and interquartile ranges (IQR), while categorical variables by frequencies and percentages.

The inter-rater (screening vs. randomization) and intra-rater (randomization vs. 4-week follow-up) agreement for the AMAT total score and for functional and endurance subscales were tested using the intraclass correlation coefficient (ICC) with 95% confidence interval (95% CI). The range of the ICC is from 0 (lowest agreement) to 1 (highest agreement).

The internal consistency of AMAT total score, functional and endurance subscales, was assessed using Cronbach’s alpha (range 0–1). Values higher than 0.70 were considered as acceptable internal consistency. Pairwise item correlations were also evaluated using Spearman correlation coefficient, considering values exceeding 0.85 as an indication for a redundant item.

Construct validity is the ability of a scale to measure the underlying theoretical construct that it is supposed to measure. Construct validity was assessed by testing convergent and divergent validity, using Spearman’s correlation coefficients between the scores of the AMAT scale (total, endurance and functional subscales) and the scores of the other functional (6MWT, SBMAFRS) and quality of life (ALSAQ-40 domains for physical mobility, ADL and independence, eating and drinking, communication, emotional reactions) scales. Convergent validity is confirmed when the correlation with scales presumed to measure the same or similar underlying construct is high. Divergent validity is confirmed when the correlation with scales presumed to measure different underlying construct is low. Both internal consistency and construct validity were evaluated considering the scores at the randomization visit.

Due to the limited prevalence of SBMA, the sample size was determined based on the total number of eligible patients available during the study period (*N* = 29). A formal sample size calculation was not performed.

## Results

A total of 29 individuals with SBMA were included in the study. The median age was 57 years (IQR 52–65, range 43–74), with a median disease duration of 4.8 years (IQR 1.2–8.4). The scores of functional and quality of life scales are shown in Table 1.

**Table 1:** Demographical and clinical characteristics of individuals with SBMA

	Median﻿	IQR﻿
Ages	57	52-65
	n	%
Age at diagnosis	51	44–57
Disease duration (years)	4.8	1.2–8.4
ARCAG repeats	44	41–46
	n	%
Muscle atrophy	27	93.1
Limb weakness	28	96.6
Bulbar palsy	29	100
	Median	IQR
Six minutes walking distance (meters)	429	287–522
SBMA-FRS total score	43	39–47
AMAT total score	36	29–40
ALSAQ-40 - physical mobility	35	13–48
ALSAQ-40 - ADL and independence	20	8–35
ALSAQ-40 - eating and drinking	17	0–42
ALSAQ-40 - communication	9	4–23
ALSAQ-40 - emotional reactions	18	8–28

The median AMAT total score was 36 (IQR 29–40), the median functional subscale score was 18 (IQR 14–19) and the median endurance subscale score was 18 (IQR 14–21).

The inter-rater ICC was 0.90 (95% CI 0.80–0.95) for the AMAT total score, 0.91 (95% CI 0.82–0.96) for the functional subscale, and 0.86 (95% CI 0.73–0.93) for the endurance subscale. The intra-rater ICC was 0.96 (95% CI 0.92–0.98) for the AMAT total score, 0.92 (95% CI 0.84–0.96) for the functional subscale, and 0.94 (95% CI 0.88–0.97) for the endurance subscale.

The Cronbach’s alpha was 0.90 for the AMAT total score, 0.87 for the functional subscale and 0.74 for the endurance subscale. Pairwise Spearman’s correlation coefficients between AMAT items ranged from 0.12 to 0.79 for the functional subscale, and from − 0.11 to 0.69 for the endurance subscale.

The correlation of AMAT total and subscales scores with the 6MWT (Spearman’s correlation coefficient 0.83–0.92) and the SBMAFRS total score (0.64–0.79) was high., with higher correlations for the endurance subscale (Table 2). A strong negative correlation of the AMAT total and subscales scores was found for the ALSAQ-40 physical mobility domain (0.80–0.87), followed by ADL and independence domain (0.68–0.81). A weak negative correlation was found with ALSAQ-40 eating and drinking (0.34–0.47) and emotional reactions domains (0.49–0.52) and with disease duration (0.49–0.57). No correlation was found with the communication domain of the ALSAQ-40 scale (Table 2).

**Table 2:** Spearman’s correlation coefficients (Rho) of AMAT total and subscales scores with functional and quality of life scales scores

	Total score		Functional subscale		Endurance subscale	
	Rho	*p*-value	Rho	*p*-value	Rho	*p*-value
Six minutes walking distance (meters)	0.92	< 0.0001	0.83	< 0.0001	0.90	< 0.0001
SBMA-FRS total score	0.78	< 0.0001	0.64	0.0002	0.79	< 0.0001
ALSAQ-40 - physical mobility	-0.87	< 0.0001	-0.80	< 0.0001	-0.85	< 0.0001
ALSAQ-40 - ADL and independence	-0.81	< 0.0001	-0.68	< 0.0001	-0.79	< 0.0001
ALSAQ-40 - eating and drinking	-0.47	0.0140	-0.34	0.0710	-0.45	0.0133
ALSAQ-40 - communication	-0.12	0.5297	-0.13	0.4783	-0.08	0.6713
ALSAQ-40 - emotional reactions	-0.52	0.0035	-0.50	0.0055	-0.49	0.0075
Disease duration	-0.57	0.0013	-0.53	0.0032	-0.51	0.0043

## Discussion

This study successfully validated the Italian version of the Adult Myopathy Assessment Tool (AMAT) for use in individuals with Spinal and Bulbar Muscular Atrophy (SBMA). Our findings demonstrate excellent psychometric properties, including strong inter- and intra-rater agreement, good internal consistency, and robust construct validity. These results support the utility of the Italian AMAT as a reliable and valid outcome measure for clinical trials and routine clinical assessment in Italian-speaking SBMA populations.

The rigorous forward- and back-translation process, involving independent translators and a consensus meeting, ensured the semantic and conceptual equivalence of the Italian AMAT to its original English version. This systematic approach is crucial for cross-cultural adaptation of assessment tools, minimizing potential biases and ensuring that the translated version accurately reflects the construct intended by the original developers.

Our inter-rater reliability analysis demonstrated an excellent agreement between different raters, confirming that the Italian AMAT can be consistently administered by trained investigators. The 30-day interval between assessments for inter-rater reliability was strategically chosen to minimize the likelihood of true clinical change while mitigating any potential training effect, thus providing a robust measure of agreement.

The internal consistency of the Italian AMAT, as assessed by Cronbach’s alpha, yielded values well above the commonly accepted threshold of 0.70 for good internal consistency. This consistency suggests that the items within the Italian AMAT effectively measure a cohesive underlying construct. Furthermore, the pairwise Spearman’s correlation coefficients between AMAT items indicate that while items are related, there is no significant redundancy (i.e., no values exceeding 0.85).

The construct validity of the Italian AMAT was strongly supported by both convergent and divergent validity analyses. We observed high correlations between the AMAT total and subscale scores and other established functional measures, such as the 6MWT and the SBMAFRS total score. These strong positive correlations confirm convergent validity, as these scales are expected to measure similar aspects of physical function in SBMA. Notably, the endurance subscale showed particularly high correlations with these functional measures, highlighting its relevance to objective physical performance.

Conversely, the Italian AMAT demonstrated appropriate divergent validity. We found a strong negative correlation with the ALSAQ-40 physical mobility domain and ADL and independence domain. This is expected, as higher scores on the AMAT (indicating better function) should correlate with lower scores on quality of life scales (indicating less impairment). Medium negative correlations were also observed with the ALSAQ-40 eating and drinking and emotional reactions domains, and with disease duration, further supporting divergent validity. Crucially, no correlation was found with the communication domain of the ALSAQ-40 scale, which is consistent with the AMAT’s focus on physical and endurance aspects rather than communication abilities in SBMA. This pattern of correlations reinforces the notion that the AMAT specifically assesses the intended constructs of impairment and functional limitations in SBMA, distinct from other aspects of quality of life not directly related to physical function.

Comparing our findings to the original validation study by Harris-Love et al. [7], our results for the Italian AMAT are remarkably consistent. The original study also reported excellent construct validity and good internal consistency (Cronbach’s alpha = 0.74–0.90), based on significant associations with strength, objective, and subjective physical performance measures, and self-reported physical status. The strong alignment of our psychometric properties with those of the original English version underscores the successful cultural and linguistic adaptation of the AMAT into Italian.

One limitation of the present study is the sample size of 29 patients, although this is a reasonable number for a rare disease like SBMA and our consecutive recruitment supports the absence of selection bias.

In conclusion, the Italian version of the AMAT scale demonstrates excellent psychometric properties and is a reliable and valid tool for assessing impairment and functional limitations in SBMA subjects. Despite limited responsiveness relative to SBMAFRS and 6MWT [12], the excellent reliability and construct validity of the scale supports its use as a secondary outcome measures in the ongoing clenbuterol clinical trial, enabling accurate and consistent assessment of therapeutic efficacy in Italian-speaking patients. Furthermore, this validated translation facilitates cross-national comparisons and collaborative research efforts, ultimately contributing to a better understanding and management of SBMA globally.

## Supplementary Information

Below is the link to the electronic supplementary material.

Supplementary Material 1

## Data Availability

Anonymized data are available from the corresponding author upon request. The dataset generated in this study is available in the Zenodo repository https://zenodo.org/communities/irfmn-irccs?q=&l=list&p=1&s=10&sort=newest .
