# Supplementary material for: Reliability and construct validity of the Italian version of AMAT scale in SBMA subjects
Source: Neurol Sci. 2026 May 2;47(5):470. doi: 10.1007/s10072-026-09052-x (PMC13135011; doi:10.1007/s10072-026-09052-x)
Supplement: Supplementary file 1 — Supplementary Material 1 [file 10072_2026_9052_MOESM1_ESM.docx]

**The Adult Myopathy Assessment Tool (AMAT)**

| 1. ^†^**Resistenza al sollevamento della testa: “Alza la testa dal lettino”. Il paziente è sdraiato supino, flette la testa e il collo in avanti e cerca di mantenere la posizione raggiunta. Il test si conclude quando la nuca tocca il lettino** | |
| --- | --- |
| 0 | <5 secondi o non in grado di effettuare il movimento |
| 1 | 5–30 secondi |
| 2 | 31–60 secondi |
| 3 | 61–90 secondi |
| 4 | >90 secondi |

| 1. **Da supino a prono**: “Girati a faccia in giù ruotando su un fianco senza fermarti, terminando con le braccia distese lungo il corpo”. Il paziente parte da posizione supina con le braccia distese lungo i fianchi; il test si conclude quando il paziente è in posizione prona con le braccia distese lungo i fianchi | |
| --- | --- |
| 0 | ≥10 secondi per effettuare il movimento o non in grado di effettuarlo |
| 1 | Movimento completato in un tempo compreso tra >6 e <10 secondi oppure se durante il movimento il paziente non ha liberato il braccio verso il quale ha effettuato la rotazione |
| 2 | Movimento completato in un tempo compreso tra >3 e ≤6 secondi |
| 3 | Movimento completato ≤3 secondi |

| 1. **Flessione modificata**: “Esegui una flessione, finendo con i gomiti il più tesi possible; le ginocchia toccheranno il lettino durante il movimento." Il paziente inizia l’esercizio con le mani rivolte verso il lettino e con le braccia in posizione 0 gradi in abduzione | |
| --- | --- |
| 0 | Non in grado di effettuare il movimento |
| 1 | Parziale estensione dei gomiti; sterno in contatto parziale con il lettino |
| 2 | Parziale estensione dei gomiti; sterno NON in contatto con il lettino |
| 3 | Completa estensione dei gomiti |

| 1. ^†^**Flessioni modificate ripetute**: “Esegui quante più flessioni possibili in 2 minuti, terminando con le braccia tese; il petto dovrebbe toccare il lettino ad ogni ripetizione”. Il paziente inizia con le mani rivolte verso il lettino e le spalle a 0 gradi in abduzione. La velocità di esecuzione viene scelta dal paziente; il test termina se il movimento viene compiuto con una tecnica errata per 2 tentativi consecutivi; i tentativi errati non devono essere conteggiati. | |
| --- | --- |
| 0 | Non in grado di effettuare il movimento |
| 1 | 1 - 10 ripetizioni |
| 2 | 11 - 20 ripetizioni |
| 3 | 21 – 30 ripetizioni |
| 4 | 31 – 40 ripetizioni |

| 1. **Sit-Up**: "Esegui un sit-up". Il test inizia dalla posizione supina con le ginocchia completamente estese e con le mani sulle cosce o sull'addome; l’esaminatore applica un contro-bilanciamento alle estremità inferiori distali per i punteggi 0-2. | |
| --- | --- |
| 0 | Non in grado di effettuare il movimento |
| 1 | Scapole e vertebra T7 non in contatto col lettino (muscolo retto dell’addome), movimento effettuato con l’ausilio di contrappeso da parte del tester |
| 2 | Vertebra L1 non in contatto col lettino, addominale completato nella sua interezza (muscoli flessori dell’anca), movimento effettuato con l’ausilio di contrappeso da parte del tester |
| 3 | Addominale completato nella sua interezza, senza l’ausilio di contrappeso da parte del tester |

| 1. ^†^**Da supino a seduto**: “Spostati in posizione seduta sul bordo del lettino il più velocemente possibile” Il paziente inizia da posizione supina, con le gambe tese e le braccia lungo i fianchi. Il test termina quando i piedi toccano il pavimento e il tronco è verticale. | |
| --- | --- |
| 0 | ≥12 secondi per effettuare il movimento o non in grado di effettuarlo |
| 1 | Movimento completato in un tempo compreso tra >7 e <12 secondi |
| 2 | Movimento completato in un tempo compreso tra >4 e ≤7 secondi |
| 3 | Movimento completato in ≤4 secondi |

| 1. **Braccia alzate**: “Allunga entrambe le braccia più in alto che puoi sopra la testa con i gomiti tesi.” Completare entro il range di movimento passivo (PROM) disponibile. Se presente asimmetria tra i due arti assegnare il punteggio all’estremità superiore più debole | |
| --- | --- |
| 0 | Non in grado di alzare le braccia a livello dell’articolazione acromion-clavicolare |
| 1 | Mani alzate tra l’articolazione acromion-clavicolare e la sommità della testa |
| 2 | Mani alzate al di sopra della sommità della testa senza estensione completa dei gomiti |
| 3 | Mani alzate al di sopra della sommità della testa con estensione completa dei gomiti |
| 1. ^†^**Resistenza al sollevamento delle braccia**: “Alza entrambe le mani in avanti all’altezza degli occhi mantenendo i gomiti dritti” (flessione della spalla). Tronco eretto senza iperestensione ed entrambi i piedi sono appoggiati al pavimento; il test si conclude se la flessione delle spalle scende sotto i 90 gradi; Se presente asimmetria tra i due arti assegnare il punteggio all’estremità superiore più debole | |
| 0 | <5 secondi o non in grado di effettuare il movimento |
| 1 | 5–30 secondi |
| 2 | 31–60 secondi |
| 3 | 61–90 secondi |
| 4 | >90 secondi |
| 1. **Da seduto ad alzato**: “Alzati con il minimo ausilio possibile delle braccia.” Il paziente è seduto sul lettino con il bordo posto a metà della coscia e il tronco è eretto, la parte inferiore delle gambe è verticale e le ginocchia a 90 gradi misurate con un goniometro. Il contatto con la parte posteriore delle gambe e il lettino non è possibile | |
| 0 | Non in grado di effettuare il movimento |
| 1 | Movimento effettuato con due o più estremità in contatto col lettino o con le cosce |
| 2 | Movimento effettuato con una estremità in contatto col lettino o con le cosce |
| 3 | Movimento effettuato senza il contatto di alcuna estremità col lettino o con le cosce |
| 1. ^†^**Resistenza alla flessione dell'anca**: “Solleva e tieni in aria il ginocchio della tua gamba dominante” Paziente seduto con anche e ginocchia a novanta gradi; senza scarpe; piede non dominante appoggiato al pavimento; tronco eretto con il bordo del lettino posto a metà della coscia; è permesso appoggiare le estremità superiori sul lettino; l’altezza di sollevamento del ginocchio si basa sulla metà del range di movimento passivo; il test termina quando il piede tocca il pavimento. | |
| 0 | <5 secondi o non in grado di effettuare il movimento |
| 1 | 5–30 secondi |
| 2 | 31–60 secondi |
| 3 | 61–90 secondi |
| 4 | >90 secondi |
| **11.** ^†^**Resistenza all'estensione del ginocchio**: “Mantieni il ginocchio della tua gamba dominante il più dritto possibile” Senza scarpe; piede non dominante appoggiato al pavimento; tronco eretto con cosce completamente appoggiate al lettino; il test si conclude quando il piede tocca il pavimento; movimento effettuato nella totalità del range di movimento passivo del paziente. | |
| 0 | <5 secondi o non in grado di effettuare il movimento |
| 1 | 5–30 secondi |
| 2 | 31–60 secondi |
| 3 | 61–90 secondi |
| 4 | >90 secondi |

| **12.** ^†^**Sollevamento ripetuto del tallone**: “Stando in piedi solo sulla gamba dominante, solleva il tallone da terra.” Senza scarpe; la velocità di esecuzione viene scelta dal paziente; il test si conclude quando la base del 5° metatarso o il mesopiede non si alzano completamente dal pavimento; il test termina se il movimento viene compiuto con una tecnica errata per 2 tentativi consecutivi; i tentativi errati non vengono conteggiati; le ginocchia rimangono il più dritte possibile senza ulteriori flessioni durante le ripetizioni; un supporto esterno minimo può essere fornito appoggiandosi al muro; i limiti sono due minuti o 30 ripetizioni. | |
| --- | --- |
| 0 | Non in grado di effettuare il movimento |
| 1 | 1–7 ripetizioni |
| 2 | 8–15 ripetizioni |
| 3 | 16–23 ripetizioni |
| 4 | 24–30 ripetizioni |
| **13. Step-up**: “Posiziona la gamba dominante sul gradino da 7 pollici,(~18 cm) e fai un passo in avanti portando anche il piede a terra sullo stesso gradino, con il minor ausilio possibile delle braccia.” Dovrebbe essere disponibile un corrimano o un supporto stabile a cui il paziente può appoggiarsi con entrambe le braccia in caso di bisogno. | |
| 0 | Non in grado di effettuare il movimento |
| 1 | Movimento effettuato con l’ausilio di entrambe le braccia |
| 2 | Movimento effettuato con l’ausilio di un braccio |
| 3 | Movimento effettuato senza l’ausilio delle braccia |
